# Anthocyanin Extracted from Black Soybean Seed Coats Prevents Autoimmune Arthritis by Suppressing the Development of Th17 Cells and Synthesis of Proinflammatory Cytokines by Such Cells, via Inhibition of NF-κB

**DOI:** 10.1371/journal.pone.0138201

**Published:** 2015-11-06

**Authors:** Hong Ki Min, Sung-Min Kim, Seung-Ye Baek, Jung-Won Woo, Jin-Sil Park, Mi-La Cho, Jennifer Lee, Seung-Ki Kwok, Sae Woong Kim, Sung-Hwan Park

**Affiliations:** 1 Rheumatism Research Center, Catholic Research Institute of Medical Science, The Catholic University of Korea, Seoul, South Korea; 2 Division of Rheumatology, Department of Internal Medicine, School of Medicine, The Catholic University of Korea, Seoul, South Korea; 3 Office of Clinical Development, Genexine Inc., Korea Bio Park, Seongnam, Gyeonggi-do, South Korea; 4 Catholic Integrative Medicine Research Institute, College of Medicine, The Catholic University of Korea, Seoul, South Korea; 5 Department of Urology, College of Medicine, The Catholic University of Korea, Seoul, South Korea; Veterans Affairs Medical Center, UNITED STATES

## Abstract

**Introduction:**

Oxidative stress plays a role in the pathogenesis of rheumatoid arthritis (RA). Anthocyanin is a plant antioxidant. We investigated the therapeutic effects of anthocyanin extracted from black soybean seed coats (AEBS) in a murine model of collagen-induced arthritis (CIA) and human peripheral blood mononuclear cells (PBMCs) and explored possible mechanisms by which AEBS might exert anti-arthritic effects.

**Material and Methods:**

CIA was induced in DBA/1J mice. Cytokine levels were measured via enzyme-linked immunosorbent assays. Joints were assessed in terms of arthritis incidence, clinical arthritis scores, and histological features. The extent of oxidative stress in affected joints was determined by measuring the levels of nitrotyrosine and inducible nitric oxide synthase. NF-κB activity was assayed by measuring the ratio of phosphorylated IκB to total IκB via Western blotting. Th17 cells were stained with antibodies against CD4, IL-17, and STAT3. Osteoclast formation was assessed via TRAP staining and measurement of osteoclast-specific mRNA levels.

**Results:**

In the CIA model, AEBS decreased the incidence of arthritis, histological inflammation, cartilage scores, and oxidative stress. AEBS reduced the levels of proinflammatory cytokines in affected joints of CIA mice and suppressed NF-κB signaling. AEBS decreased Th17 cell numbers in spleen of CIA mice. Additionally, AEBS repressed differentiation of Th17 cells and expression of Th17-associated genes *in vitro*, in both splenocytes of naïve DBA/1J mice and human PBMCs. *In vitro*, the numbers of both human and mouse tartrate-resistant acid phosphatase^+^ (TRAP) multinucleated cells fell, in a dose-dependent manner, upon addition of AEBS.

**Conclusions:**

The anti-arthritic effects of AEBS were associated with decreases in Th17 cell numbers, and the levels of proinflammatory cytokines synthesized by such cells, mediated via suppression of NF-κB signaling. Additionally, AEBS suppressed osteoclastogenesis and reduced oxidative stress levels.

## Introduction

Rheumatoid arthritis (RA) is the most common form of autoimmune inflammatory arthritis, and affects about 1% of the world’s population [1]. The principal problems associated with RA are systemic inflammation and progressive destruction of diarthrodial joints [2]. Joint destruction is the most important cause of functional impairment in RA patients.

Since methotrexate has been introduced as a RA treatment agent, several conventional disease-modifying anti-rheumatic drugs (DMARDs) have been used to treat RA. Conventional DMARDs not only relieve the signs and symptoms of acute RA-associated inflammation but also prevent radiographic progression of the disease [3]. Furthermore, several biological DMARDs, including tumor necrosis factor (TNF)-α blockers, the anti-cluster of differentiation (CD) 20 monoclonal antibody, and a fusion protein consisting of the cytotoxic T-lymphocyte-associated protein-4 linked to the Fc region of immunoglobulin (Ig) G, have been used to treat RA. Such drugs exhibited therapeutic effects in RA patients refractory to conventional DMARDs [4–8]. However, not all RA patients respond to biological DMARDs in terms of reductions in systemic inflammation and retardation of joint destruction. Therefore, novel therapeutic agents are required to treat RA patients refractory to existing drugs.

Type-17 helper T (Th17) cells are one of major helper T cells involved in the pathogenesis and progression of RA [9,10]. Proinflammatory cytokines including interleukin (IL)-6 and TNF-α trigger naive CD4^+^ T cell differentiation into Th17 cells [11], and also play direct roles in RA pathogenesis [2]. Recently, drugs suppressing Th17 activities, and inhibiting the expression of pathological cytokines by such cells, have become candidates for RA treatment [12–14].

The pathogenesis of RA is not fully understood, but several factors are known to play important roles. Oxidative stress is one such factor, and indeed aggravates RA [15]. Excessive oxidative stress induced autoimmune arthritis in a mouse model, and RA patients exhibit increased levels of oxidative stress, as compared to healthy controls [16,17]. Antioxidants exert dual functions, of which one is removal of excessive reactive oxygen species (ROS), and the other reduction of inflammation. Both the antioxidative and anti-inflammatory functions are mediated by activation of nuclear factor E2-related factor 2 [18]. Therefore, an emerging concept is that antioxidants may ameliorate RA symptoms and severity by reducing the inflammatory response.

Anthocyanin is a representative antioxidant of the flavonoid family found in plant tissues. Black soybean seed coats are an excellent source of anthocyanin [19]. Anthocyanin extracted from black soybean seed coat (AEBS) exerted anti-inflammatory effects in a rat model of urogenital inflammatory disease [20, 21]. In addition, anthocyanin exerts immunomodulatory effects via inhibition of signaling by the nuclear factor kappa-light-chain-enhancer of activated B cells (NF-κB) [22]. The anti-inflammatory effects of AEBS in patients with inflammatory arthritis have not yet been proven.

NF-κB is expressed in various types of animal cells, including immune cells, and controls DNA transcription. NF-κB signaling is stimulated by several factors including bacterial antigens, free radicals, and oxidized low-density lipoproteins. NF-κB signaling regulates both innate and adaptive immunity [23] and, thus, influences the pathogenesis of autoimmune diseases [24].

Thus, we hypothesized that AEBS would ameliorate inflammatory arthritis and prevent joint destruction in a mouse model of collagen-induced arthritis (CIA) by suppressing oxidative stress and exerting immunomodulatory effects on Th17 cells. To identify the mechanisms of such actions, we explored how AEBS affected Th17 cell development, expression of proinflammatory cytokines, and osteoclast formation.

## Material and Methods

### Animals

Male DBA/1J mice aged 4–6 weeks were purchased from Orient Bio (Seongnam, South Korea) and fed food and water ad libitum. All animals were allowed to acclimatize for 2 weeks prior to commencement of experiments. The animals were housed under specific pathogen-free conditions and were used at 7–10 weeks of age. All experimental procedures were evaluated and approved by the Animal Research Ethics Committee of the Catholic University of Korea.

### Induction and evaluation of CIA

DBA/1J mice, 7 weeks of age, were intradermally immunized (Day 0) at the base of the tail with 100 μg amounts of bovine type II collagen (CII; Chondrex Inc., Redmond, WA) suspended in complete Freund’s adjuvant (Mycobacterium tuberculosis (H37Ra) 4mg/ml; CFA, Chondrex Inc). To explore the effects of AEBS on CIA, DBA/1J mice were fed daily for 7 weeks with 60 mg/kg AEBS (dissolved in saline, or saline as a control), commencing on day 7 after primary immunization and keep feeding for 7 weeks, and were monitored for a total of 8 weeks. Mice were visually examined three times per week, and development of arthritis in the peripheral joints recorded. The arthritis score index used to record disease severity was as follows: 0 = no evidence of erythema or swelling; 1 = erythema and mild swelling confined to the mid-foot (the tarsals) or ankle joints; 2 = erythema and mild swelling extending from the ankle to the mid-foot; 3 = erythema and moderate swelling extending from the ankle to the metatarsal joints; and, 4 = erythema and severe swelling encompassing the ankle, foot, and digits. The maximum possible score per mouse was 16. All scoring was performed by two independent observers blinded to the identities of the experimental and control groups.

### Preparation of AEBS

AEBS used in present experiment was offered from the Rural Development Administration, Suwon, Republic of Korea. The anthocyanin was extracted from Cheongja 3 black soybean seed coats. Detailed procedures of extracting anthocyanin were described in previous study [21]. AEBS was composed of the following three components: cyanidin-3-O-glucoside (68.3%), delphinidin-3-O-glucoside (25.2%), and petunidine-3-Oglucoside (6.5%). AEBS was dissolved in saline and fed to the mice.

### Histopathological analysis

Joint tissues were fixed in 4% (v/v) paraformaldehyde, decalcified in a histological decalcifying agent (Calci-Clear Rapid; National Diagnostics, Atlanta, GA), embedded in paraffin, and sectioned. The sections were stained with hematoxylin-eosin (H&E), safranin O, and toluidine blue (to detect proteoglycans). H&E-stained sections were scored in terms of inflammation and bone erosion. Inflammation was scored using the following criteria: 0 = no inflammation, 1 = slight thickening of the lining or infiltration of some cells into the underlying layer; 2 = slight thickening of the lining with infiltration of some cells into the underlying layer, 3 = thickening of the lining, with an influx of cells into the underlying layer, and cells evident in the synovial space; and, 4 = extensive infiltration of the synovium by inflammatory cells. Cartilage damage was evaluated by staining with safranin-O and toluidine blue, and the extent of such damage scored using the following criteria: 0 = no destruction; 1 = minimal erosion (limited to single spots); 2 = slight-to-moderate erosion in a limited area; 3 = more extensive erosion; and, 4 = general destruction.

### Measurement of IgG subtype levels

Blood was taken from the orbital sinuses of AEBS- and saline-treated mice and the sera stored at −20°C prior to analysis. Total IgG, IgG1, and IgG2a antibody levels were measured in 100,000-fold dilutions of sera using a mouse total IgG, IgG1, and IgG2a enzyme-linked immunosorbent assay (ELISA) quantitation kit (Bethyl Lab Co., Montgomery, TX). Optical densities (ODs) were measured at 450 nm with the aid of an ELISA plate reader (Bio-Rad, Hercules, CA).

### Murine T cell isolation and differentiation

Mouse spleen cells were obtained from naïve DBA/1J mice and sieved through a mesh and red blood cells (RBCs) lysed in hypotonic ACK buffer (0.15 mM NH_4_Cl, 1 mM KCO_3_, and 0.1 mM EDTA; pH 7.4). To purify splenic CD4^+^ T cells, splenocytes were incubated with CD4-coated magnetic beads and isolated on magnetic-activated cell sorting (MACS) separation columns (Miltenyi Biotec, Seoul, South Korea). Naïve cells were collected from this population by selecting for CD4^+^CD62L^high^CD25^low^CD44^low^ cells (>97% purity; Dako Cytomation, Glostrup, Denmark) and were also obtained using CD4^+^CD62L^+^ magnetic beads (Miltenyi Biotec). To induce Th17-polarization, splenocytes were stimulated with plate-bound anti-CD3 (145-2C11) (0.5 μg/ml; BD Pharmingen, San Jose, CA), anti-CD28 (37.51) (1 μg/ml; eBiosciences, San Diego, CA), anti-interferon-γ (37895) (IFN-γ; 2 μg/ml), anti-IL-4 (30340) (2 μg/ml), recombinant human (rh) TGF-β (CHO cell derived) (2 ng/ml; all from Pepro-Tech, Rocky Hill, NJ), and IL-6 (E.coli derived) (20 ng/ml; R&D Systems, Minneapolis, MN) for 72 h. Th0 cells were stimulated with only anti-CD3 and anti-CD28, in the absence of added cytokines. Cells were cultured at RPMI (5% FBS, 1% antibiotics). Total ribonucleic acid (RNA) was extracted from splenocytes by using the Tri reagent (Molecular Research Center, Cincinnati, OH). Supernatant IL-17 levels were assayed. Cells were pretreated with AEBS (50–200μg/ml) for 1 day and washed. Then harvested cells were seeded at culture media with adding AEBS (50–200μg/ml) under the aforementioned Th17-polarizing conditions.

### Human CD4^+^ T cell isolation and differentiation

Human peripheral blood mononuclear cells (PBMCs) were obtained from normal healthy volunteers. Cells were separated from buffy coats via Ficoll-Hypaque density gradient centrifugation (Amersham Biosciences, Uppsala, Sweden). RBCs were lysed in hypotonic ACK buffer. CD4^+^ T cells were isolated from PBMCs using a CD4^+^ T cell isolation kit (Miltenyi Biotec) according to the manufacturer’s instructions. To induce Th17-polarization, CD4^+^ T cells were stimulated with plate-bound anti-CD3 (0.5 μg/ml), anti-CD28 (1 μg/ml; BD Pharmingen), anti-IFN-c (2 μg/ml), anti-IL-4 (2 μg/ml), IL-1β (20 ng/ml), and IL-6 (20 ng/ml; R&D systems) for 72 h. Cells were cultured at RPMI (10% FBS, 1% antibiotics). Total RNA was extracted using the Tri reagent (Molecular Research Center) according to the manufacturer’s instructions. The supernatants were assayed in terms of IL-17 content. Cells were pretreated with AEBS (50–200μg/ml) for 1 day and washed. Then harvested cells were seeded at culture media with adding AEBS (50–200μg/ml) under the aforementioned Th17-polarizing conditions. The written informed consents were obtained from each participant after providing enough information and explanation about procedure. This study was approved by the Seoul St. Mary’s Hospital Institutional Review Board.

### TRAP staining

Tartrate-resistant acid phosphatase (TRAP) staining was performed with the aid of a commercial kit (Sigma-Aldrich) according to the manufacturer’s instructions, omitting counterstaining with hematoxylin. TRAP-positive multinucleate cells with three or more nuclei were considered to be osteoclasts. All histological assessments were made by three independent observers blinded to treatment group.

### *In vitro* osteoclastogenesis

Bone marrow-derived monocytes/macrophages (BMM) were isolated from the tibiae and femurs of naïve DBA/1J mice and incubated in minimum essential medium-alpha (α-MEM; Invitrogen, Burlingame, CA) containing antibiotics and 10% (v/v) heat-inactivated fetal bovine serum, for 12 h, to separate floating from adherent cells. Floating cells were seeded into 48-well plates at 5 X 10^5^ cells/well and cultured in the presence of 10 ng/ml rh macrophage colony-stimulating factor (M-CSF; R&D Systems, Minneapolis, MN) in α-MEM. Three days later, washed nonadherent cells and preosteoclasts were further cultured in the presence of 10 ng/ml M-CSF and 50 ng/ml Receptor Activator of Nuclear Factor κB ligand (RANKL; Peprotech, London, UK), and various concentrations of AEBS, for 4 days, to generate osteoclasts. PBMCs were prepared from normal healthy volunteers and separated from buffy coats via Ficoll-Hypaque (GE Healthcare, Uppsala, Sweden) chromatography. PBMCs were separated from RBCs, seeded into 24-well plates at 5 X 10^5^ cells/well, and incubated at 37°C for 2 h to separate floating from adherent cells. The adherent cells were washed with sterile phosphate buffered saline and cultured in the presence of 100 ng/ml M-CSF. After 3 days, the cells were further incubated with 25 ng/ml M-CSF, 30 ng/ml RANKL, and various concentrations of AEBS, for 9 days. On day 3, the medium was replaced with fresh medium containing M-CSF, RANKL, and AEBS. Informed consent was obtained from all participating subjects. The study protocol was reviewed and approved by our Institutional Review Board that evaluates human studies.

### Enzyme-linked immunosorbent assay (ELISA)

Antibodies against mouse IL-17 and biotinylated anti-mouse IL-17 (R&D Systems) served as capture and detection antibodies, respectively. The fluorescent substrate horseradish peroxidase-avidin (R&D Systems) was used for color development. The levels of cytokines in test samples were determined by reference to standard curves constructed using serial dilutions of recombinant IL-17 (R&D Systems).

### Immunohistochemistry

Tissues were first incubated with primary antibodies against Nitrotyrosine (39B6), IL-17 (H-132), IL-1 β (H-153), IL-6 (M-19), and TNF-α (M-18) (Santa Cruz Biotechnology, Santa Cruz, CA), iNOS (Abcam, Cambridge, MA), overnight at 4°C. Incubation with a biotinylated secondary antibody followed and, finally, a streptavidin-peroxidase complex was added and incubation continued for a further 1 h. Final colored products were developed using the chromogen diaminobenzidine (Thermo Scientific, Rockford, IL) and the sections examined under a photomicroscope (Olympus, Tokyo, Japan). The cells showing positive IL-17, IL-6, TNF-α, IL-1β, iNOS, and nitrotyrosine were enumerated visually at higher magnification (projected on a screen) by four individuals, and the mean values are presented.

### Confocal microscopy

For confocal staining, 7 μm-thick sections of spleens were stained using Alexa Fluor^®^ 488 conjugated anti-CD4 (GK1.5) (BioLegend, San Diego, CA), phycoerythrin (PE)-conjugated anti-IL-17 (eBio17B7), allophycocyanin (APC)- conjugated STAT3 (M59-50) (eBiosciences, San Diego, CA), phycoerythrin (PE)-conjugated p-STAT3 Y705 (4/p-STAT3), and S727 (49/p-STAT3) (all from BD PharMingen, San Diego, CA), allophycocyanin (APC)- conjugated CD25 (PC61) (BioLegend), phycoerythrin (PE)-conjugated anti-Mouse/Rat forkhead box P3 (Foxp3) (FJK-16s) (eBiosciences), phycoerythrin (PE)-conjugated Mouse anti-STAT5 (pY694) (47/Stat5(pY694) (BD PharMingen), STAT5 (3H7) Rabbit mAb (Cell Signaling), and PE donkey anti-rabbit IgG (Poly4064) (BioLegend). Stained sections were examined under a microscope (LSM 510 Meta; Carl Zeiss, Oberkochen, Germany) at 400x magnification.

### Western blotting

Splenocytes extracted from AEBS- and vehicle-treated CIA mice were lysed in Halt lysis buffer containing Halt phosphatase inhibitor (Therm Pierce), and centrifuged for 15 min at 14,000 g at 4°C. Proteins were separated via 10% sodium dodecyl sulfate-polyacrylamide gel (Amresco) electrophoresis and transferred to Hybond ECL membranes (GE Healthcare) for Western blotting analysis using the SNAP i.d. protein detection system (Millipore, Billerica, MA). Blots were incubated with antibody against the inhibitor of κB (44D4)(IκB; 1:1,000, Cell Signaling), phosphorylated IκB (14D4)(p-IκB; 1:1,000, Cell Signaling), and β-actin (AC-15)(1:2,000, Sigma) for 10 min at room temperature. After washing, horseradish peroxidase-conjugated secondary antibodies were added and incubated for 10 min at room temperature. After further washing, bands were detected using an ECL detection kit (Pierce, Rockford, IL) and Hyperfilm (Agfa, Mortsel, Belgium).

### Intracellular staining and flow cytometry

The intracellular levels of cytokines and transcription factors were assessed via staining with anti-CD4-PerCPCy5.5 (RM4-5) and anti-IL-17-FITC (eBio17B7)(eBioscience). In brief, cells were stimulated for 4 h with phorbol myristate acetate (PMA; 25 ng/ml) and ionomycin (250 ng/ml) in the presence of GolgiStop. The cells were next labeled for 30 min and permeabilized using Cytofix/Cytoperm solution (BD Pharmingen). Events were collected and analyzed with the aid of FlowJo software (Tree Star, OR).

### Real-time polymerase chain reaction (PCR)

PCR amplification and analysis were performed with the aid of a Light-Cycler 2.0 (Roche Diagnostics, Mannheim, Germany) running software version 4.0. All reactions were performed using LightCycler FastStart DNAmaster SYBR green I (Takara, Otsu, Japan), according to the manufacturer’s instructions. The following primers were used to amplify mouse sequences: matrix metalloproteinase 9 (MMP-9), 5′–CTGTCCAGACCAAGGGTACAGCCT–3′ (sense), 5′–GAGGTATAGTGGGACACATAGTGG–3′ (antisense); TRAP, 5′–TCCTGGCTCAAAAAGCAGTT–3′ (sense), 5′–ACATAGCCCACACCGTTCTC–3′ (antisense); calcitonin receptor, 5′–CGGACTTTGACACAGCAGAA–3′ (sense), 5′–AGCAGCAATCGACAAGGAGT– 3′ (antisense); nuclear factor of activated T-cells, cytoplasmic 1 (NFATc1), 5′–CGGGAAGAAGATGGTGCTGT–3′ (sense), 5′–TTGGACGGGGCTGGTTAT–3′ (antisense); OSCAR, 5′–CCTAGCCTCATACCCCCAG–3′ (sense), 5′–CAAACCGCCAGGCAGATTG–3′ (antisense); IL-17, 5′–CCTCAAAGCTCAGCGTGTCC–3′ (sense), 5′–GAGCT CACTTTTGCGCCAAG–3′ (antisense); aryl hydrocarbon receptor (AHR), 5′-GCAGCTCACTTTGGATGACA–3′ (sense), 5′—CCAAACGTCACAGGACATTG–3′ (antisense); and β-actin, 5′– GTACGACCAGAGGCATACAGG–3′ (sense), 5′–GATGACGATATCGCTGCGCTG– 3′ (antisense). The following primers were used for human sequences: TRAP, 5′–GACCACCTTGGCAATGTCTCTG– 3′ (sense), 5′–TGGCTGAGGAAGTCATCTGAGTTG– 3′ (antisense); IL-17A, 5′–CAACCGATCCACCTCACCTT –3′ (sense), 5’–GGCACTTTGCCTCCCAGAT–3′ (antisense); RORc, 5′– AGTCGGAAGGCAAGATCAGA –3′ (sense), 5′–CAAGAGAGGTTCTGGGCAAG–3′ (antisense); cathepsin K, 5′–TGAGGCTTCTCTTGGTGTCCATAC–3′ (sense), 5′–AAAGGGTGTCATTACTGCGGG–3′ (antisense); MMP-9, 5′–CGCAGACATCGTCATCCAGT–3′ (sense), 5′–GGATTGGCCTTGGAAGATGA–3′ (antisense); Receptor Activator of Nuclear Factor κB (RANK), 5′–GCTCTAACAAATGTGAACCAGGA–3′ (sense), 5′–GCCTTGCCTGTATCACAAACT–3′ (antisense); and β-actin, 5′–GGACTTCGAGCAAGAGATGG–3′ (sense), 5′–TGTGTTGGCGTACAGGTCTTTG–3′ (antisense). Messenger RNA (mRNA) expression levels were normalized to those of mRNAs encoding β-actin.

### Statistical analysis

Results were calculated using the GraphPad Prism 5.1 software and are presented as the means ± SD of at least three experiments. A *P* value of < 0.05 was considered to indicate statistical significance. Data were compared by two-way ANOVA with Bonferroni’s post-test and Student’s *t*-test, as appropriate. The ratio of p-IκB was obtained by using Quantity one software.

## Results

### AEBS attenuates the development and progression of arthritis in CIA mice

**Fig 1:**
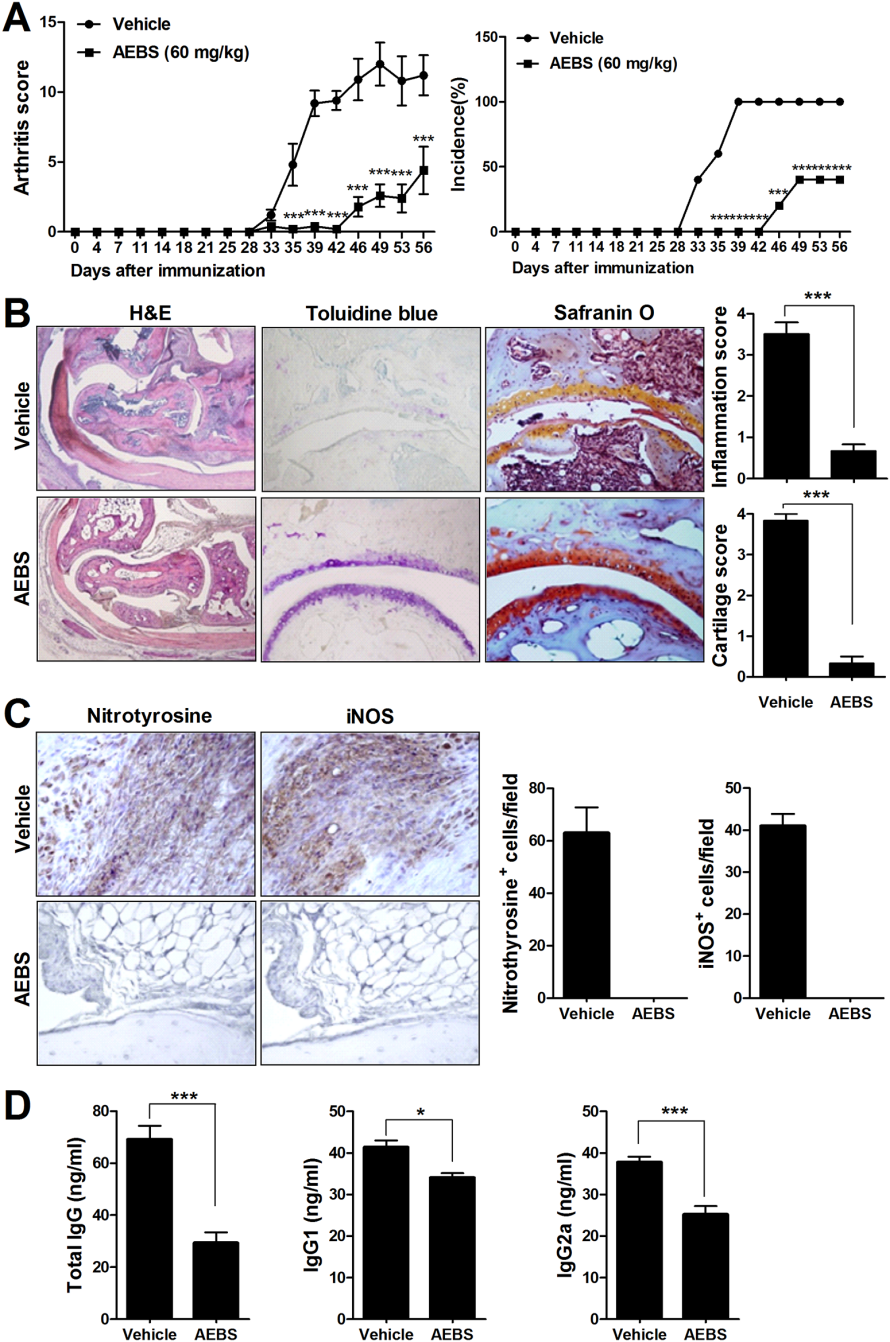
AEBS suppresses inflammatory arthritis in CIA mice. Both the arthritis score and the incidence of arthritis were reduced in CIA mice given AEBS. Mice were immunized with CII. Seven days later, mice were given oral AEBS (60 mg/kg) or vehicle, daily, for 8 weeks. (A) The mean arthritis scores ± SDs (left panel) and arthritis incidence scores (right panel). (B) Histological data on joints from CIA mice given AEBS or vehicle. Mice were sacrificed on day 49 after immunization. Joint tissue sections were stained with H&E, toluidine blue, and safranin O. Joints from mice given AEBS exhibited mild erosive arthritis, whereas those from vehicle-treated mice exhibited markedly erosive destructive arthritis. Representative photographs are shown. Original magnifications ×40 or ×200, as indicated. The histological scores (measuring inflammation and the extent of cartilage damage) in mice given AEBS (n = 5) or vehicle (n = 5) are shown in the right graph. Data are expressed as means ± SDs. ***P < 0.01 compared to the vehicle-treated control group. (C) Tissue sections from joints of CIA mice given AEBS or vehicle were stained with anti-nitrotyrosine and anti-iNOS antibodies. Cells stained with either antibody are brown in color. Original magnification ×400. The cells showing positive nitrotyrosine, and iNOS were enumerated visually at higher magnification (projected on a screen) by four individuals, and the mean values are presented (cells/field). (D) Levels of total circulating IgG, IgG1, and IgG2a in CIA mice given AEBS (n = 8) or vehicle (n = 8). Total IgG, IgG1, and IgG2a levels were determined in sera of individual mice via ELISA. Data are expressed as means ± SDs. * *P* < 0.05, *** *P* < 0.001 compared to the vehicle-treated group. Each experiment was performed 3 times.

To explore the effects of AEBS on CIA development and progression, DBA/1J mice were immunized with CII and treated with AEBS. Oral AEBS (60 mg/kg) given daily for 7 weeks, commencing 7 days after primary immunization, reduced the arthritis score and disease incidence in AEBS-treated mice, as compared to controls (Fig 1A). Consistent with the arthritis score data, staining with safranin O and toluidine blue showed that cartilage destruction was less evident in AEBS-treated than vehicle-treated mice (Fig 1B). In terms of oxidative stress, joints from AEBS-treated mice had lower levels of nitrotyrosine and inducible nitric oxide synthase (iNOS) than did vehicle-treated mice (Fig 1C). Also, AEBS reduced the serum levels of total IgG, IgG1, and IgG2a (Fig 1D). These results suggest that AEBS suppressed induction of arthritis, oxidative stress, and joint destruction in CIA mice.

### Suppression of proinflammatory cytokine synthesis and IκB phosphorylation by AEBS

**Fig 2:**
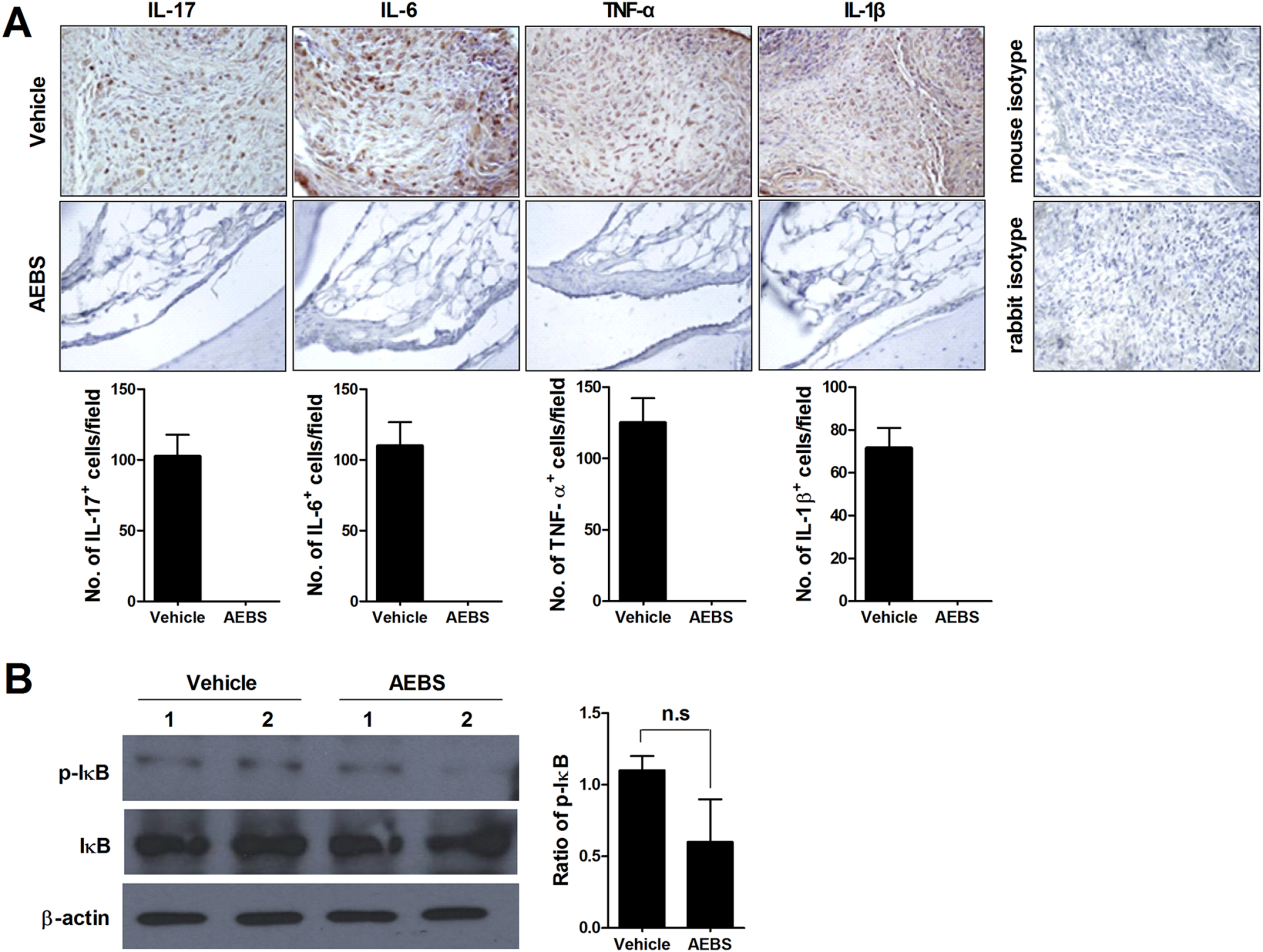
AEBS reduces Th17 cell numbers in CIA mice. Tissues were obtained and stained when the mice were sacrificed (8weeks from immunization). (A) AEBS reduced the expression levels of IL-17, IL-6, TNF-α, and IL-1β in synovial tissues. Tissue sections from joints of CIA mice given AEBS (n = 5) or vehicle (n = 5) were stained with anti-IL-17, anti-IL-6, anti-TNF-α, and anti-IL-1β antibodies; or anti-isotype control antibodies. Stained cells are brown in color. Original magnification ×400. The cells showing positive IL-17, IL-6, TNF- α, and IL-1β were enumerated visually at higher magnification (projected on a screen) by four individuals, and the mean values are presented (cells/field). (B) Pooled splenocytes from CIA mice given AEBS were cultured with PMA (25 ng/ml) and ionomycin (250 ng/ml) for 30 min and p-IκB levels determined via Western blotting. Data are expressed as means ± SDs. *P < 0.05 compared to the vehicle-treated group. Each experiment was performed 3 times.

Expression of proinflammatory cytokines was assessed by staining affected joints with specific antibodies detecting IL-1β, IL-6, IL-17, and TNF-α, and enumerating the cell populations synthesizing such cytokines. To explore activation of NF-κB signaling, we measured the levels of IκB and p-IκB. AEBS-treated CIA mice had lower levels of IL-1β, IL-6, IL-17, and TNF-α in affected joints than did vehicle-treated mice, and the cell populations synthesizing such cytokines were also reduced in test animals (Fig 2A). The p-IκB:IκB ratio was non-significantly lower in the AEBS-treated group than in controls (Fig 2B). These results suggest that AEBS reduced inflammation of affected joints, the numbers of cells secreting proinflammatory cytokines, and trend to suppress NF-κB signaling.

### AEBS reduces the STAT3-Th17 cell population in spleens of CIA mice

**Fig 3:**
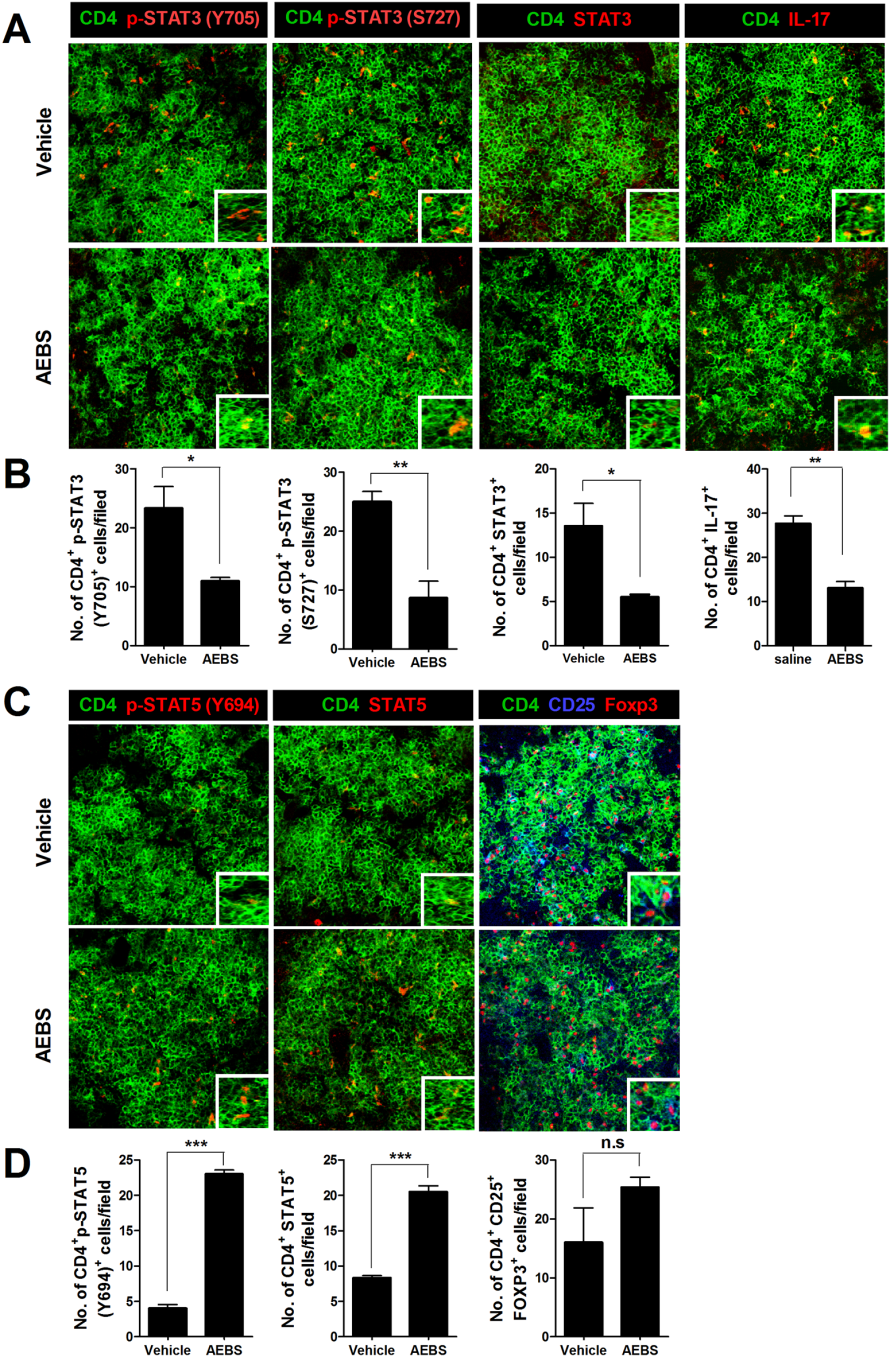
AEBS decreases Th17 cell numbers in CIA mice. (A) Spleens from each mouse were stained for CD4^+^p-STAT3(Y705)^+^, CD4^+^p-STAT3(S727)^+^, CD4^+^STAT3^+^, and CD4^+^IL-17^+^ T cells using antibodies specific for CD4, STAT3Y705, STAT3S727, and IL-17. The cell populations were analyzed via laser confocal microscopy (original magnification ×400). (B) The numbers of T cells positive for CD4^+^STAT3Y705^+^, CD4^+^STAT3S727^+^, CD4^+^STAT3^+^, and CD4^+^IL-17^+^ in each mouse were visually counted at a higher magnification (after projection of fields onto a screen) and mean values are shown. (C) Spleens from each mouse were stained for CD4^+^p-STAT5(Y694)^+^, CD4^+^STAT5^+^, and CD4^+^CD25^+^Foxp3^+^. (D) The numbers of T cells positive for CD4^+^p-STAT5(Y694)^+^, CD4^+^STAT5^+^, and CD4^+^CD25^+^Foxp3^+^ in each mouse were visually counted at a higher magnification (after projection of fields onto a screen) and mean values are shown. (Number of AEBS treated mice = 5, Number of vehicle treated mice = 5) Data are expressed as means ± SDs. *P < 0.05, **P < 0.01. Each experiment was performed 3 times.

To explore whether AEBS could control Th17 cell populations, we extracted spleens from AEBS-treated and control CIA mice and examined spleens via confocal microscopy. Th17 cells were fewer in number in AEBS-treated than control mice (Fig 3A). The numbers of CD4^+^p-STAT3(Y705)^+^, CD4^+^p-STAT3(S727)^+^, CD4^+^STAT3^+^, and CD4^+^IL-17^+^ cells in the AEBS-treated group were significantly lower than in the control group (Fig 3B). The number of CD4^+^p-STAT5(Y694)^+^, and CD4^+^STAT5^+^ cells were significantly higher in AEBS-treated group (Fig 3C and 3D). CD4^+^CD25^+^Foxp3^+^ cell counts were higher in AEBS-treated group, but the result was not significant (Fig 3C and 3D).

### Inhibition of Th17 cell differentiation and expression of Th17-related genes in DBA/1J mice and humans

**Fig 4:**
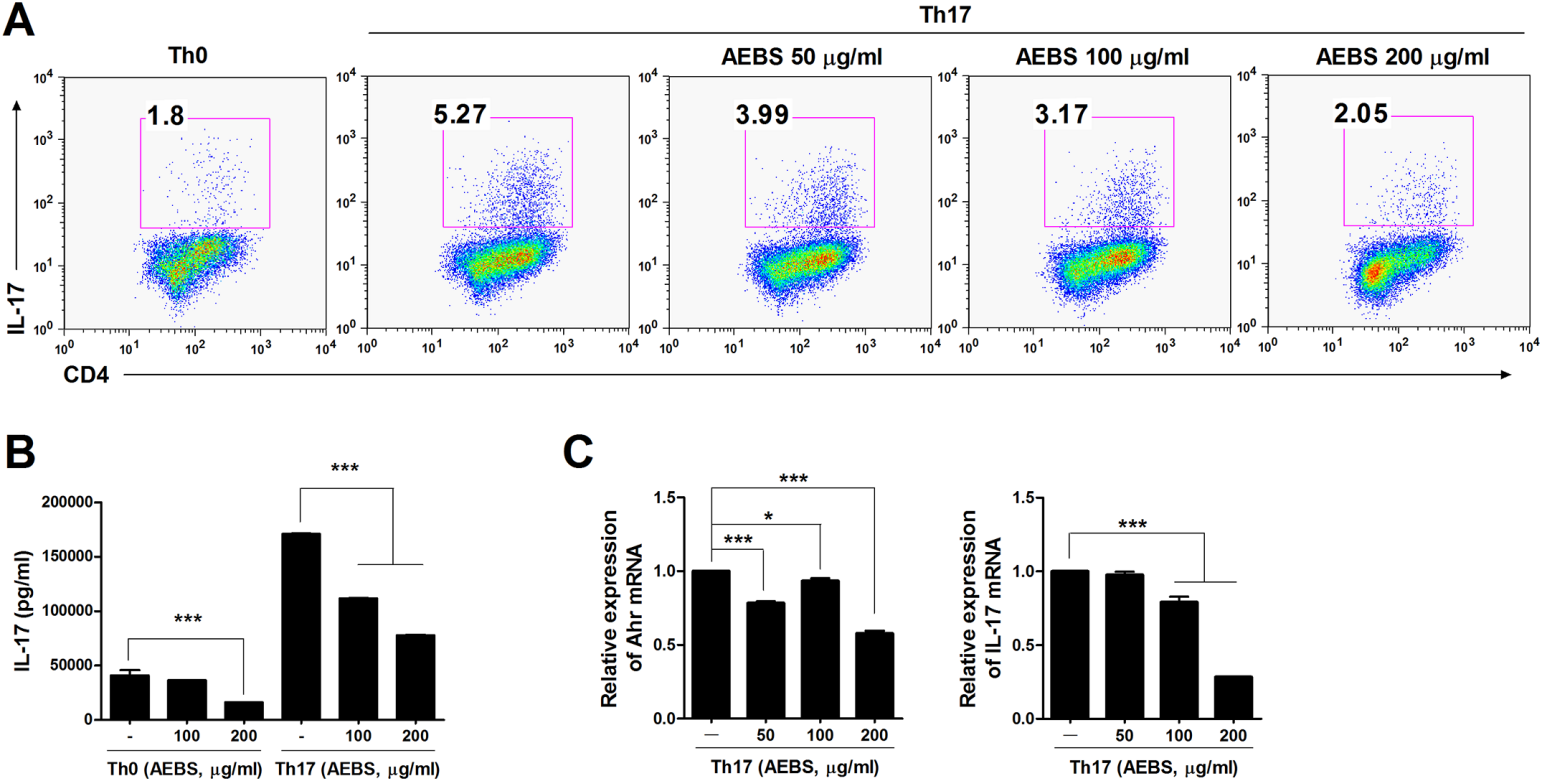
AEBS represses IL-17 synthesis by mouse CD4^+^ T cells. (A) CD4^+^ T cells isolated from the spleens of naïve DBA/1J mice were cultured under Th17-polarizing conditions in the presence or absence of AEBS (50–200μg/ml). Three days later, the cells were stained with antibodies against CD4 and IL-17, as described in Materials and Methods. (B) The IL-17 levels in the culture supernatants described in (A) above were measured via ELISA. (C) The levels of *IL-17* and *Ahr* mRNAs were determined by real-time PCR. (Number of AEBS treated mice = 3, Number of vehicle treated mice = 3) Each experiment was performed 3 times.

**Fig 5:**
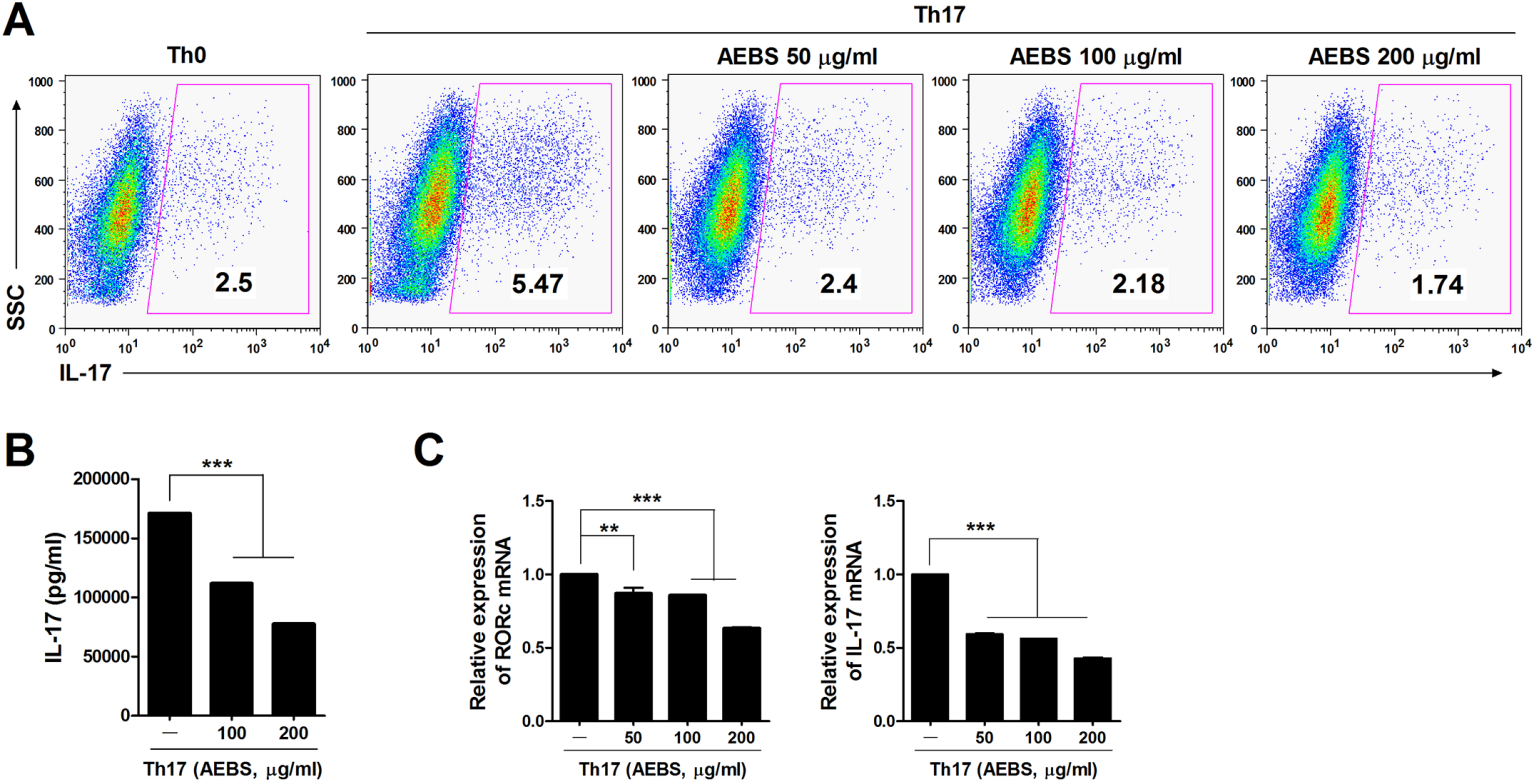
AEBS represses IL-17 synthesis by human PBMCs. (A) CD4^+^ T cells isolated from PBMCs of normal healthy volunteers were cultured under Th17-polarizing conditions in the presence or absence of AEBS (50–200μg/ml). Three days later, the cells were stained with antibodies against CD4 and IL-17, as described in the Materials and Methods section. The proportion of Th17 cells were measured by Side-scattered light (SSC) method. (B) The levels of IL-17 in the culture supernatants described in (A) above were measured using sandwich ELISA (R&D Systems, Minneapolis, MN). (C) The levels of *IL-17* and *RORc* mRNAs were determined using real-time PCR. (Number of AEBS group = 3, Number of vehicle group = 3) Each experiment was performed 3 times.

CD4^+^ T cells were isolated from splenocytes of naïve DBA/1J mice and human PBMCs and cultured under Th17-polarizing conditions for 3 days with or without AEBS (50–200 μg/ml) and analyzed at day 3. The IL-17 levels in culture supernatants were measured. AEBS dose-dependently reduced the proportions of IL-17-expressing T cells in CD4^+^ cell populations of both the spleens of DBA/1J mice and human PBMCs (Figs 4A and 5A). IL-17 levels in culture supernants were reduced in AEBS-treated DBA/1J mice and humans (Figs 4B and 5B). Furthermore, expression of Th17 cell differentiation-associated genes, including *Ahr* and *IL-17* of DBA/1J mice and *RORc* and *IL-17* of humans, was suppressed by AEBS (Figs 4C and 5C). These results suggest that AEBS suppressed differentiation of Th17 cells and the expression of Th17 cell differentiation-associated genes.

### AEBS inhibits *in vitro* osteoclastogenesis of DBA/1J mice BMM and human PBMC

**Fig 6:**
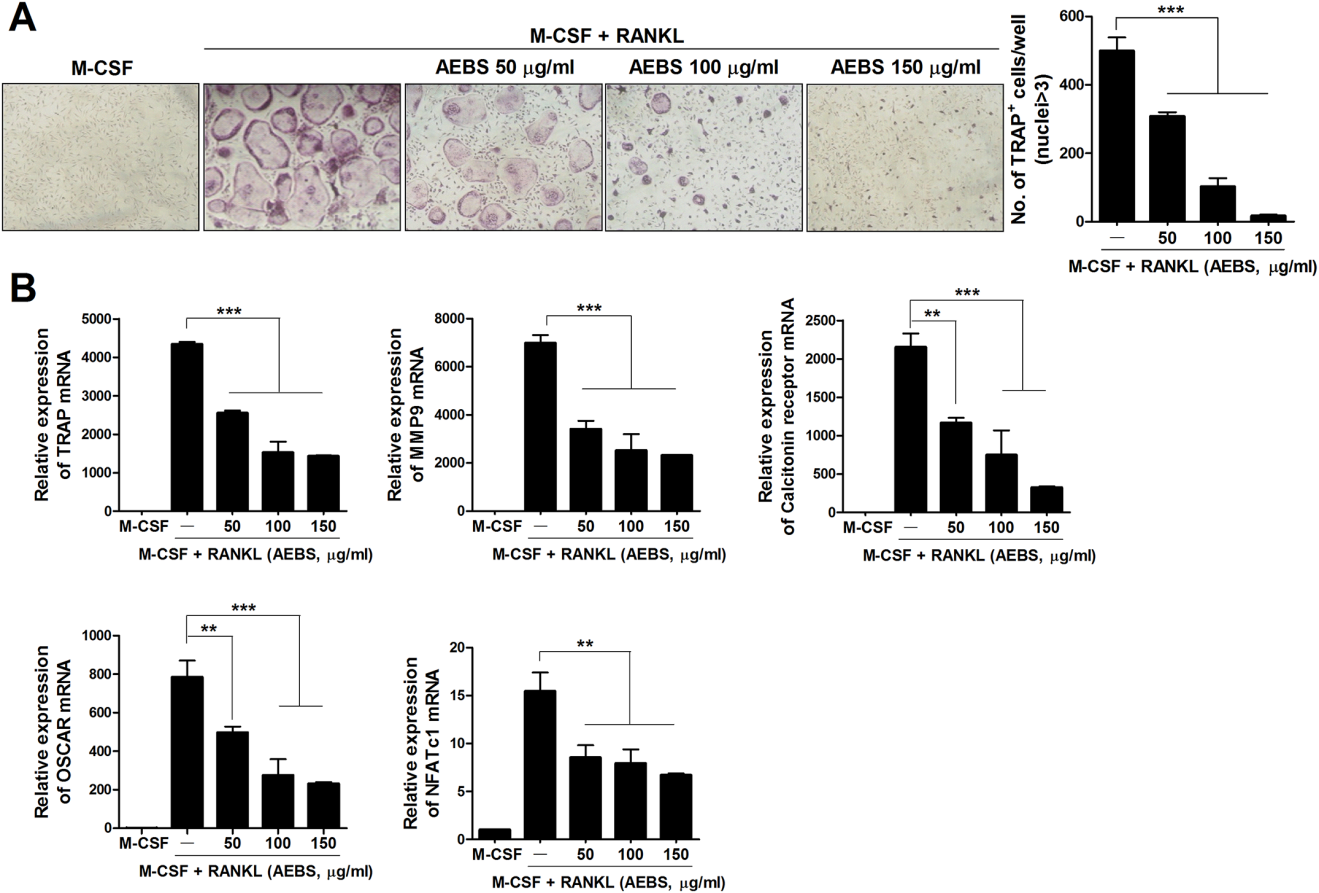
AEBS inhibits osteoclast formation in DBA/1J mice. (A) *In vitro*, AEBS inhibited osteoclast formation in a dose-dependent manner. BMM cells from naïve DBA/1J mice treated with vehicle were cultured in the presence of M-CSF (10 ng/ml), and/or RANKL (50 ng/ml), and/or AEBS (50–150 μg/ml). The medium was changed every 2 days. After 7 days, cells were stained to detect TRAP activity. Original magnification ×100. ***P < 0.001. (B) The levels of mRNAs encoding the osteoclastogenic markers MMP-9, the calcitonin receptor, TRAP, OSCAR, and NFATc1 were measured via real-time PCR under the experimental conditions described in (A) above. **P < 0.01, ***P < 0.001. (Number of AEBS treated mice = 3, Number of vehicle treated mice = 3) Each experiment was performed 3 times.

**Fig 7:**
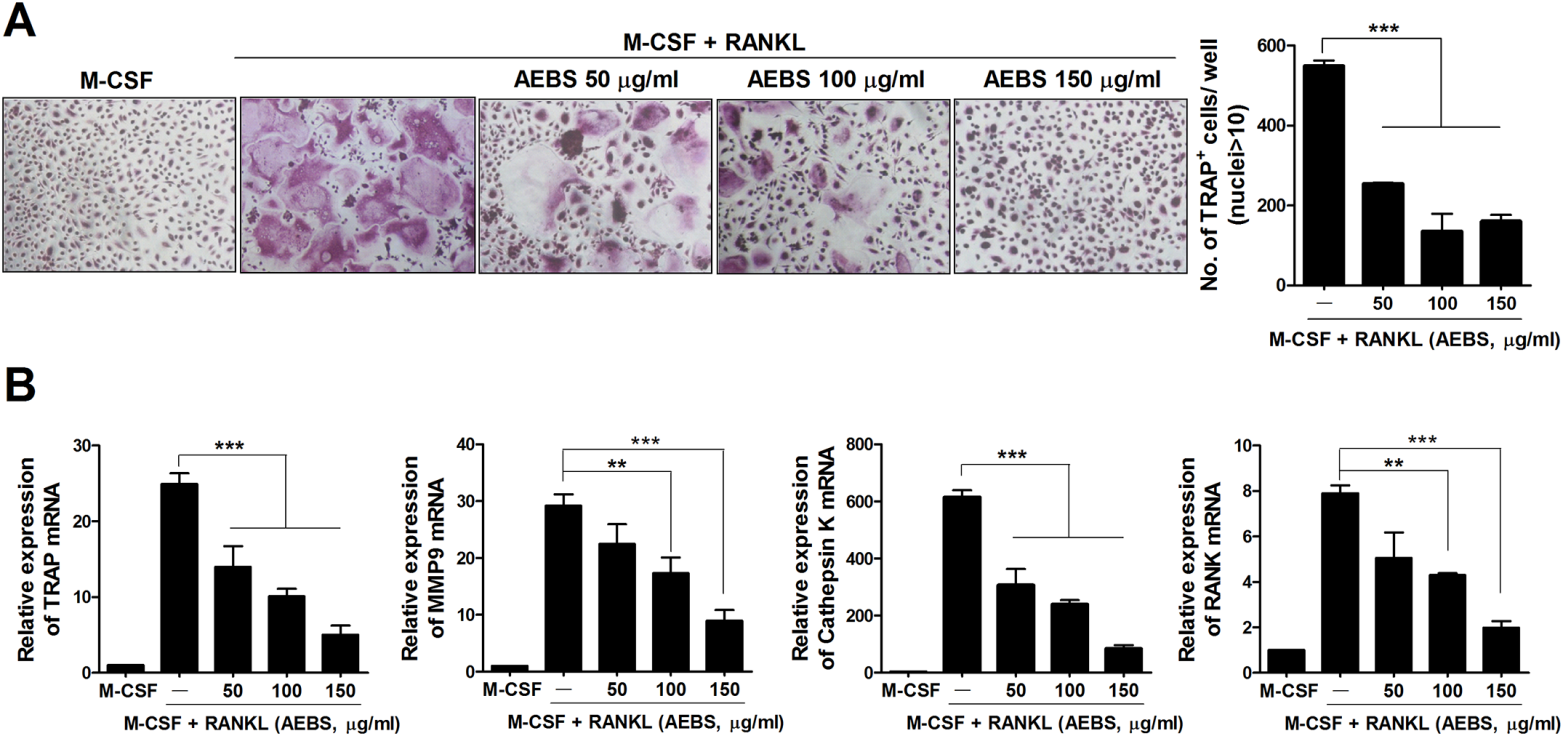
AEBS inhibits the differentiation of human monocytes into osteoclasts. (A) AEBS inhibited osteoclast formation, in a dose-dependent manner, in the presence of M-CSF and RANKL. Human monocytes from healthy volunteers (n = 3) were cultured in the presence of M-CSF (25 ng/ml), and/or RANKL (30 ng/ml), and/or AEBS (50–150 μg/ml). After 9 days, cells were stained to detect TRAP activity. Representative photographs from each group are shown in the left panels. The numbers of multinucleated TRAP^+^ cells are shown in the right panel. Original magnification ×100. ***P < 0.001. (B) The levels of mRNAs encoding TRAP, MMP-9, and cathepsin K were quantified via real-time PCR. **P < 0.01, ***P < 0.001, compared to treatment with M-CSF and RANKL. (Number of AEBS group = 3, Number of vehicle group = 3) Each experiment was performed 3 times.

To explore the effects of AEBS on osteoclastogenesis, we cultured naïve DBA/1J mice BMM and human monocytes with M-CSF and RANKL in the presence of 50, 100, or 150 μg/mL AEBS. The numbers of TRAP^+^ multinucleated cells were lower in AEBS-treated groups than in controls (Figs 6A and 7A). The levels of mRNAs encoding TRAP, MMP-9, the calcitonin receptor, OSCAR, and NFATc1 were significantly reduced in AEBS-treatedgroup (Fig 6B). Likewise, the levels of mRNAs encoding human osteoclast-associated genes, including TRAP, MMP-9, cathepsin K, and RANK, were lower in the AEBS than the control groups (Fig 7B). These results indicate that AEBS suppresses osteoclastogenesis in both DBA/1J mice and humans.

## Discussion

In the present study, we showed that AEBS exerted therapeutic effects in a RA mouse model both *in vivo* and *in vitro*; and in humans *in vitro*. Three probable mechanisms by which AEBS exerts anti-arthritic effects were revealed. One of major mechanisms is suppression of Th17 differentiation and production of proinflammatory cytokines via AEBS-induced inhibition of NF-κB signaling. Another likely mechanism is reduction of oxidative stress in affected joints. The final probable mechanism is inhibition of osteoclastogenesis via downregulation of osteoclast-associated genes. Decendit A et al. have previously revealed the beneficial effects of anthocyanin on RA rat model mainly on suppressing macrophage, oxidative stress, and proinlfammatory cytokines [25]. The present study clarified the beneficial effects of anthocyanin on autoimmune arthritis by Th17 suppression, reducing proinflammatory cytokines by modulating NF- κB signalling, and inhibition of osteoclast for the first time.

Over the past decade or so, Th17 cells have been identified as distinct subtypes of CD4^+^ cells within both the Th1 and Th2 cell groupings [9]. Th17 cells are characterized by the production of various proinflammatory cytokines including IL-6, IL-17, IL-21, and TNF-α, and play crucial roles in the pathogenesis of various autoimmune diseases, including RA [26, 27]. Naive precursor CD4^+^ cells develop into Th17 cells when exposed to proinflammatory cytokines including IL-1, IL-6, and IL-23 [27]. Such cytokines are responsible not only for Th17 differentiation but also contribute to the structural damage and systemic inflammation associated with RA [14, 27]. Therefore, effective control of Th17 differentiation and proinflammatory cytokine production is an attractive approach to the management of RA. In the present study, AEBS showed beneficial effects on development of CIA by suppression of Th17 differentiation and inhibition of proinflammatory cytokine production *in vivo* of CIA mice and *in vitro of* DBA/1J mice.

Several transcription factors play roles in the differentiation of naive CD4^+^ cells into Th17 cells. Recently, AHR activation has been revealed to be a co-factor involved in Th17 differentiation and the production of cytokines by such cells [28, 29]. The *RORc* gene encodes both RORγ and RORγt, transcription factors essential for Th17 cell differentiation and IL-17 expression [30]. Thus, inhibition of Th17-associated genes including *Ahr* and *RORc* would be therapeutically valuable, in that Th17 differentiation would be suppressed and production of Th17-associated proinflammatory cytokines reduced. In the present study, we showed that AEBS suppressed the some of the genes associated with differentiation toward the Th17 lineage, and inhibited IL-17 expression.

Regulatory T cell (Treg) is known to play opposite role of Th17 in autoimmune diseases including RA [11]. STAT-5 is the major transcription factor which promotes naive CD4^+^ T cell to differentiate into Treg. In present study, increase of Treg count was not significant in AEBS-treated group, AEBS activated STAT-5 signalling significantly. Aforementioned results support the possibility of AEBS on regulating Treg, and further researches are needed to clarify effects of AEBS on modulating Treg and STAT-5 signalling.

NF-κB is a major transcription factor controlling the expression of many genes involved in proinflammatory cytokine production. In the repressed state, NF-κB is inactivated by binding to IκB, which occludes the NF-κB DNA-binding cleft [31]. NF-κB activation is initiated by phosphorylation of IκB, and proinflammatory cytokine production is then induced. The *RORc* gene, essential for Th17 differentiation, is a target of NF-κB [32]. Reactive oxygen species (ROS) are known to activate NF-κB signaling [33]. Thus, the data, taken together, suggest that a reduction in oxidative stress would mitigate inflammatory arthritis by inhibiting NF-κB signaling. In turn, this would reduce proinflammatory cytokine expression and that of Th17- associated genes including *RORc*. In the present study, although the p-IκB:IκB ratios between AEBS- and vehicle-treated group were not significant, AEBS showed a trend to attenuated NF-κB signaling. Further researches are required to clarify the beneficial effects of AEBS on NF-κB signalling.

Joint destruction is the principal cause of functional impairment in RA patients. Thus, therapeutic approaches seek not only to reduce systemic inflammation but also to inhibit the development of structural deformities. Osteoclasts are the cells principally responsible for joint destruction, and interaction of RANK with RANKL is essential for osteoclastogenesis [2, 34, 35]. Various cytokines including IL-1, IL-6, IL-17, and TNF-α promote the formation, activity, and survival of osteoclasts [2, 35]. AEBS repressed osteoclastogenesis, the expression of osteoclast-associated genes, and osteoclastogenic cytokines.

In conclusion, oral AEBS demonstrated anti-arthritic effects *in vivo*, and relevant activities were also evident *in vitro*, using the CIA mouse model. AEBS reduced Th17 cell numbers both *in vitro* and *in vivo*, and effectively inhibited expression of proinflammatory cytokines in a CIA model by reducing NF-κB signaling. AEBS also suppressed oxidative stress in CIA mice *in vivo*, and reduced human Th17 cell populations *in vitro*. Finally osteoclastogenesis was suppressed by AEBS in both DBA/1J mice and human cells *in vitro*. Thus, we have described some of the therapeutic mechanisms of AEBS acting to mitigate autoimmune arthritis. In future, oral AEBS may provide additive beneficial effects when used to treat inflammatory arthritis, including RA, in which oxidative stress plays an important pathogenic role.
